# Assessment of Special Care Newborn Units in India

**DOI:** 10.3329/jhpn.v29i5.8904

**Published:** 2011-10

**Authors:** Sutapa Bandyopadhyay Neogi, Sumit Malhotra, Sanjay Zodpey, Pavitra Mohan

**Affiliations:** ^1^Indian Institute of Public Health–Delhi, Public Health Foundation of India, New Delhi, India; ^2^United Nations Children's Fund, India Country Office, New Delhi, India

**Keywords:** Cross-sectional studies, Neonatal mortality, Newborn care, Performance evaluation, India

## Abstract

The neonatal mortality rate in India is high and stagnant. Special Care Newborn Units (SCNUs) have been set up to provide quality level II newborn-care services in several district hospitals to meet this challenge. The units are located in some remotest districts where the burden of neonatal deaths is high, and access to special newborn care is poor. The study was conducted to assess the functioning of SCNUs in eight rural districts of India. The evaluation was based on an analysis of secondary data from the eight units that had been functioning for at least one year. A cross-sectional survey was also conducted to assess the availability of human resources, equipment, and quality care. Descriptive statistics were used for analyzing the inputs (resources) and outcomes (morbidity and mortality). The rate of mortality among admitted neonates was taken as the key outcome variable to assess the performance of the units. Chi-square test was used for analyzing the trend of case-fatality rate over a period of 3-5 years considering the first year of operationalization as the base. Correlation coefficients were estimated to understand the possible association of case-fatality rate with factors, such as bed:doctor ratio, bed:nurse ratio, average duration of stay, and bed occupancy rate, and the asepsis score was determined. The rates of admission increased from a median of 16.7 per 100 deliveries in 2008 to 19.5 per 100 deliveries in 2009. The case-fatality rate reduced from 4% to 40% within one year of their functioning. Proportional mortality due to sepsis and low birthweight (LBW) declined significantly over two years (LBW <2.5 kg). The major reasons for admission and the major causes of deaths were birth asphyxia, sepsis, and LBW/prematurity. The units had a varying nurse:bed ratio (1:0.5-1:1.3). The bed occupancy rate ranged from 28% to 155% (median 103%), and the average duration of stay ranged from two days to 15 days (median 4.75 days). Repair and maintenance of equipment were a major concern. It is possible to set up and manage quality SCNUs and improve the survival of newborns with LBW and sepsis in developing countries, although several challenges relating to human resources, maintenance of equipment, and maintenance of asepsis remain.

## INTRODUCTION

Every year, four million newborn babies die in the first month of life—99% in lowand middle-income countries (1). India carries the single largest share (around 25-30%) of neonatal deaths in the world. Neonatal deaths constitute two-thirds of infant deaths in India; 45% of the deaths occur within the first two days of life (2).

It has been estimated that about 70% of neonatal deaths could be prevented if proven interventions are implemented effectively with high coverage (3). It was further estimated that health facility-based interventions can reduce neonatal mortality by 23-50% in different settings (3). Facility-based newborn care, thus, has a significant potential for improving the survival of newborns in India.

Three levels of neonatal care are envisaged. Newborn-care corners are established at every level to provide essential care at birth, including resuscitation. Level I care includes referral of sick newborns from Primary Health Centres (PHCs) to higher centres and care at Neonatal Stabilization Units (NSUs) in the first referral units. Care in the NSUs includes stabilization of sick newborns and care of low-birthweight (LBW) babies not requiring intensive care. Level II care includes functioning of Special Care Newborn Units (SCNUs) at the district hospital level. These units are equipped to handle sick newborns other than those who need ventilatory support and surgical care. The level III units are the neonatal intensive care units.

Community-based neonatal interventions aim for increased referral of sick newborns to facilities, largely to be catered by these SCNUs, thus acting as a powerful intervention to transfer the value of both community and facility-based healthcare. These units are essentially equipped with radiant warmers, phototherapy units, oxygen concentrators, pulse oxymeters, and intravenous infusion pumps, enough to treat and take care of babies with birth asphyxia, jaundice, sepsis, and LBW. These units cater to both inborn and outborn sick neonates. The recommended nurse:bed ratio is 1:1.2 while the doctor:bed ratio should be 1:4. It has been estimated that around 15-20% of all newborns require level II care in rural settings (4).

While until recently there has been little evidence of feasibility and effectiveness of level II newborn care in rural settings, recent experiences have shown that a rural district hospital can provide level II newborn care. An SCNU was set up within a public hospital in 2003 and managed by a non-profit organization in Purulia, an underdeveloped district in the state of West Bengal, India. It was demonstrated that strengthening of secondary-level care can lead to significant reduction in mortality among admitted newborns and was further estimated to lead to reduction in neonatal mortality of the entire district (5). The neonatal mortality rate (NMR) among admitted newborns reduced by 14% in the first year and by 21% in the second year after the SCNU became functional. At the population level, this was estimated to have led to reduction in the NMR by about 10% in the district in two years.

Subsequently, the SCNUs have been scaled up in many rural districts of the country. The United Nations Children's Fund (UNICEF) provided technical and financial support during the initial phase in establishing eight units. Within 3-4 years, more than 150 SCNUs were set up all over India. An amount of Rs 40-60 lac was spent to establish a single unit (6). Support from the Government is a key determinant behind the successful running of these units.

We conducted an evaluation of the initial eight units to assess the feasibility and effectiveness of such an approach in improving newborn care in rural hospitals and to understand the operational bottlenecks that affect their effectiveness.

## MATERIALS AND METHODS

The evaluation was carried out in eight units across eight states of India over a 12-month period. Those states where the SCNUs were started at the district hospitals in the last five years were identified. Within these states, the index units (the first one in the state) were selected. The units functional for less than a year were excluded. The included units were: Purulia (West Bengal) established in 2003, Mayurbhanj (Orissa) and Port Blair (Andaman and Nicobar Islands) initiated in 2006, Guna (Madhya Pradesh) established in 2007, Tonk (Rajasthan**),** Lalitpur (Uttar Pradesh), Dibrugarh (Assam), and Vaishali (Bihar) established in 2008.

Based on an extensive literature review, different components of evaluation were identified. For assessment, standards laid out by the National Neonatology Forum for accreditation of level II units in India and those adapted for the SCNUs were adopted (6). A structured instrument was prepared to capture secondary data. The tool was finalized in consultation with the National Neonatology Forum, UNICEF, and selected experts. Quantitative information was gathered on the resource inputs provided and available with the unit and performance of the units in terms of the performance on neonatal mortality and morbidity indicators.

Information was collated from the monthly reports of the SCNUs. Where incomplete or inconsistent, the records (admission registers and stock registers) were reviewed. The research team visited all the units to gather the missing information and triangulate the data with personal observations and interaction with the unit staff.

Data were collected for the 2006–September 2009 period from the eight units. Information was collected on the following parameters: number of admissions; availability of human resources (doctors and nurses); adequacy and availability of essential equipment, such as radiant warmers, phototherapy units, weighing machines, oxygen concentrators, generator, and air conditioners and their functional status; availability and adequacy of beds; maintenance of asepsis; and morbidity profile and mortality rate among the admitted newborns. Rates of admission were calculated taking the total number of livebirths in the hospitals as the denominator. The performance of each unit was assessed using case-fatality rate (CFR) (proportion of deaths among babies admitted to the SCNU) as the outcome variable. Data of the first year were taken as the baseline for every unit since data of the preceding years were not available. The CFR for the following years were compared with the baseline data. For identifying the factors that potentially affect the performance, Spearman's rank correlation was used. The factors assessed were: bed:doctor ratio, bed:nurse ratio, average duration of stay, bed occupancy rate, and asepsis score for each unit. The asepsis score was a composite score ascertained from the following factors after giving appropriate weights: 24-hour running water (2), presence of an elbow-operated wash basin (1), availability of soap (1), practice of handwashing before entering the SCNU (2), practice of handwashing after touching every baby (2), practice of wearing gowns in the SCNU (1), practice of wearing slippers in the SCNU (1), and practice of wearing mask and caps in the SCNU (1). The parameters included in the indicators were based on the observations made by the research team at the time of visit. To overcome a possible bias, the information was triangulated with the feedback obtained from the beneficiaries. At least four beneficiaries from each district were interviewed. The mothers were encouraged to narrate the instructions given before entering the SCNU and their adherence to those instructions. Data were entered in Microsoft Excel Office 2007. The Epi Info software (version 3.5.1) and Excel were used for analysis of data.

### Ethical approval

The proposal was reviewed by the Technical Review Committee of the Public Health Foundation of India, and ethical clearance was obtained. Permission was sought from the concerned authorities (Civil Surgeon of the hospital/programme officers and SCNU-in-charge) to collect information, after informing them of the purpose of the study.

## RESULTS

### Background characteristics

All the SCNUs are located in the remote and underdeveloped districts of the states (Table 1) (7,8). The units were established with support from the UNICEF and state governments and were fully functional for at least one year at the time of the evaluation. The number of deliveries in 2008 and 2009 in the district hospitals exceeded 5,000 per year (range 2,115-9,289, median 6,446). The proportion of stillbirths ranged from 1.3 to 6 for 100 deliveries, and the prevalence of low birthweight ranged from 20% to 30% across the units in 2009.

**Table 1.** Demographic indicators of districts where SCNUs surveyed are located (7,8)

District (state)	Infant mortality rate per 1,000 livebirths	% of literacy rate of women	% of institutional deliveries	Sex ratio (no. of females per 1,000 males)
India*	57	53.7	40.5	933
Tonk (Rajasthan)*	93	32.2	25.8	934
Dibrugarh (Assam)*	45	53.2	33.3	923
Mayurbhanj (Orissa)*	61	37.84	32.4	980
Purulia (West Bengal)*	56	59.0	48.9	954
Lalitpur (Uttar Pradesh)*	100	33.3	23.9	884
Vaishali (Bihar) **	68	36.6	28.2	920
Guna (Madhya Pradesh)*	107	43.06	29.8	885
Port Blair (Andaman and Nicobar Islands)*	54	76.6	78.6	844

* Source: District-level health survey—Reproductive and Child Health-II, 2002-2004;

** Source: District-level health survey—Reproductive and Child Health-III, 2007-2008;

SCNUs=Special Care Newborn Units

The number of admissions for every 100 deliveries progressively increased since the setting up of the units. On average, the rate of admissions increased from 16.7% in 2008 to 19.5% in 2009 across the units. The majority (60%) of the admitted neonates across the SCNUs were males (range 50-76% of all admissions). Most admissions were those born in the same hospital (intramural), constituting 54-91% of the total number of admissions in 2009. Outborn admissions (extramural) varied from 9% in Port Blair to 46% in Lalitpur. Around 40% of admitted babies (n=6,595) weighed 1,500-2,499 g, and another 6.5% weighed 1,000-1,499 g (Table 2).

**Morbidity profiles of neonates admitted to SCNUs**

**Fig. 1.**
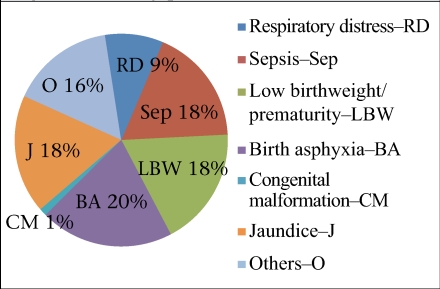
Morbidity profile of admissions in 2009

A considerable variation existed in the proportion of the burden of different conditions. For example, in Guna (n=1,616), Vaishali (n=240), and Tonk (n=1,130), respiratory distress was reported among 25-30% of babies admitted while in Mayurbhanj it was not listed among the causes. However, across the units, the neonates admitted with prime morbidities included asphyxia, sepsis, and LBW/prematurity (Fig. 1).

### Performance of SCNUs

The performance was based on the analysis of inputs (in terms of infrastructure, human resources, and equipment) and output in terms of the average duration of stay, bed occupancy rate, and aseptic measures followed. The CFR was the key outcome variable that correlated with the input and output variables.

**Table 2.** Profiles of newborns admitted to SCNUs in 2009

Name of unit	Total no. of admissions (2008)	Admission/ 100 deliveries	Inborn cases/100 admissions	No. of males/100 admissions	Proportion of newborns with birthweight of 1,500-2,499 g/100 admissions	Proportion of newborns with birthweight of <1,500 g
Tonk	1,315	34.0	65.7	70.0	44.2	11.1
Dibrugarh	1,376	26.0	90.3	50.0	40.6	9.1
Mayurbhanj	1,425	28.9	65.8	56.8	27.9	2.5
Purulia	413	4.1	71.3	64.0	44.8	4.5
Lalitpur	211	15.8	54.0	68.4	NA*	NA*
Vaishali	414	4.2	60.2	75.7	22.6	11.1
Guna	1,762	27.2	53.7	68.1	45.5	12.1
Port Blair	713	39.3	87.5	57.7	31.7	5.8
Total	7,629	19.5	68.5	63.8	38.7	8.5

Birthweight was not recorded in 40% of babies;

NA=Not applicable;

SCNUs=Special Care Newborn Units

### Inputs

#### Infrastructure

Each SCNU was unique in its layout and suffered from its own space constraints, making it difficult to adhere to the norms. Three of the eight units were close to the labour rooms while the remaining units were at a distance. While six of the eight units had a step down room, five had a designated space for breastfeeding. There are recommendations that at least 50 sq ft per bed should be available for baby care and another 50 sq ft per bed for ancillary space. Only two units complied with these recommendations (6).

We estimated the required numbers of beds for special care based on two methods (Table 3). In the first method, the average number of livebirths in the district hospital (as obtained from the respective districts), the proportion of livebirths requiring special care (15%), and the average duration of stay (7 days) were considered. In the second method, the average number of livebirths (as obtained from the respective districts), the proportion of livebirths requiring special care, and the duration of stay as obtained from the present study were used. The actual numbers of beds in the units were less than the required numbers of beds in both the instances.

#### Human resources

Three of the eight units had less number of nurses than the recommended nurse:bed ratio of 1:1.2, and three had less number of doctors than the recommended doctor:bed ratio of 1:4 (6) (Table 4). More than 80% (n=20) of the 24 doctors were paediatricians. In two units, doctors were posted exclusively for the SCNUs. In the remaining six units, doctors had to manage outdoor/indoor patients and had to attend to paediatric emergency duties, in addition to managing the SCNUs. Most staff members (21 of 24 doctors and 83 of 96 nurses) had been imparted practical training, although the duration of training varied from one day to 15 days. There was no formal refresher training.

**Table 3.** Estimation of bed requirements in Special Care Newborn Units (4)

No. of units	Existing no. of beds	No. according to NNF estimates	NNF formulae with data from study
Tonk	12	20	33
Dibrugarh	17	23	25
Mayurbhanj	12	25	27
Purulia	14	32	19
Lalitpur	12	25	13
Vaishali	13	32	3
Guna	20	32	45
Port Blair	14	10	22

NNF=National Neonatology Forum

**Table 4.** Key input and output variables to assess the performance of SCNUs

Name of unit	Total no. of beds	Nurse: bed ratio	Doctor: bed ratio	Reported time (months) for repair of essential equipment (warmers, phototherapy units)	Asepsis score (out of 11)	Average duration (days) of stay	Bed occupancy rate (%)
Tonk	12	1:1.5	1:4.0	6	5	5.1	106
Dibrugarh	17	1:0.8	1:5.7	1.5	8	4.4	137
Mayurbhanj	12	1:1.2	1:4.0	6	3	4	155.3
Purulia	14	1:0.7	1:4.7	1.5	9	15	100
Lalitpur	12	1:2	1:6.0	3	5	3.4	52
Vaishali	13	1:1	1:2.6	1.5	5	2.2	28.1
Guna	20	1:2	1:6.7	0.5	7	5.5	130
Port Blair	14	1:1.8	1:7.0	6	9	5.6	96

Seventy-five percent (18 of 24) of the doctors, although on permanent roll, were transferred from the PHCs. On the other hand, almost 68% of the nurses were hired on a contractual basis in the surveyed SCNUs.

#### Equipment

All the SCNUs had most of the essential equipment. However, once the equipment had a crash, the repair was often delayed. The breakdown time varied from one week to over six months for essential equipment across the units. In many units, by the time the SCNU was completely taken over by the Government, the warranty period of the equipment had expired. At the time of the survey, annual maintenance contract (AMC) was made in four of the eight units for essential equipment, such as baby warmers and phototherapy units. However, whether or not the unit had an AMC did not seem to influence the breakdown time.

### Output

#### Average length of stay and bed occupancy rate

These parameters were dependent to a large extent on the admission load, numbers of beds, demand for empty beds, and profiles of babies admitted to the SCNUs. High bed occupancy resulted in sharing of beds by 2-3 babies in some instances. The bed occupancy rate also indicated the burden on the nurses since each baby requiring admission in the SCNU would need special attention. During the assessment, it ranged from 28% to 155% (median 103%) across all the units. The average duration of stay in the surveyed units ranged from two days to 15 days (median 4.75 days).

#### Aseptic practices

The research team observed the components included in the composite score for asepsis and scored based on a pretested instrument. Of a maximum score of 11, the score of the units ranged from 3 to 9 (median 6).

### Outcome

The CFR varied from 7% to 18% in various units in 2009. It declined in the first year of the functioning in six of the eight SCNUs. The decrease in the first year of operations ranged from 4% to 50% (Table 5). The decline after two years (taking the first year as the baseline) ranged from 8% to 63% in the four units which were functional for three years. There was, however, a reversal in the trend observed in two of four units during the second year of their functioning. The declining trend in the CFR since the time of inception was significant for Purulia (χ^2^ for trend=24.3, p<0.001) and Guna (χ^2^ for trend=4.71, p=0.03). The proportion of babies leaving against medical advice had shown a decline in most units in 2009, ranging from 0% to 10.5%.

**Fig. 2.**
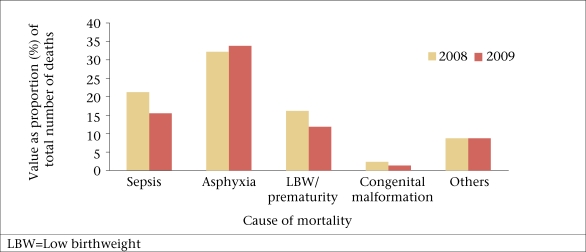
Proportional mortality rate due to sepsis, asphyxia, and low birthweight in 2008 and 2009

Asphyxia (47%), sepsis (22%), and LBW (17%) were the major causes of mortality in the units. Results of an analysis of two years of all the units, except Lalitpur (since the data on causes of deaths from Lalitpur were not reliable), showed that there was a slight decrease in proportionate mortality due to sepsis and LBW while that of asphyxia showed a modest increase (Fig. 2). However, it should be interpreted with caution since there were differences in the case definitions used by reporting units. Results of analysis of cause-specific mortality in each unit revealed that the proportional mortality rate due to sepsis and LBW decreased uniformly while that due to asphyxia increased. The decline was significant for sepsis (χ^2^=6.32, p=0.01) and LBW/prematurity (χ^2^=4.47, p=0.03) while the rise for asphyxia was not (χ^2^=0.47, p=0.49). The possibility of a bias was limited in this case since there was very little turnover of doctors, and hence, an inter-observer variation was likely to be less.

**Table 5.** Case-fatality rate in SCNUs and its decline

Name of unit	Case-fatality rates (for every 100 admissions)				Decline in mortality rate between 1 ^st^ year and 2 ^nd^ year	Decline in mortality rate between 2 ^nd^ year and 3 ^rd^ year
	2006	2007	2008	2009		
Tonk	NA	NA	7.4	7.0	4.6	NA
Dibrugarh	NA	NA	5.9	7.9	-35.0	NA
Mayurbhanj	NA	17.2	12.3	15.7	28.5	-28.2
Purulia	25.8	9.6	6.8	7.3	48.1	29.4
Lalitpur	NA	NA	16.6	18.1	-8.6	NA
Vaishali	NA	NA	21.7	12.4	43.0	NA
Guna	NA	20.6	13.8	12.0	32.9	13.6
Port Blair	NA	9.2	7.7	7.1	16.2	-0.1

NA=Not applicable;

SCNUs=Special Care Newborn Units

### What can influence performance of SCNUs?

#### Human resources

Around 14% of the variation in the CFR could be explained by the number of nurses (r^2^=0.14, 95% CI −0.21,-0.49). This should, however, be viewed with caution because the sample-size was too small to come to a conclusion. This figure remained unchanged when the total number of nurses was adjusted based on their qualifications. Interestingly, the CFR did not correlate with the number of doctors.

#### Aseptic practices

The aseptic practice score strongly correlated with the CFR. Nearly 50% of the variation in the CFR across the units could be explained by the aseptic practices observed by the doctors and nurses in the units (r^2^=0.495, 95% CI 0.09-0.89). Although the number of the observed units was small, a strong correlation and plausibility of the association suggest that the aseptic practices do critically determine the outcomes of newborns in the SCNUs. The practices followed were triangulated with the practices as narrated by the beneficiaries. Interestingly, the mothers at Purulia and Port Blair could explicitly speak about the aseptic measures. Not only that, they even expressed that those practices continued even after they were discharged from the hospital. They appreciated the importance of following those measures meticulously.

#### Average duration of stay

Case fatality reduced with the increased duration of stay. Around 22% of the variation in the CFR could be explained by the average duration of stay.

### Proportion of very LBW babies

The proportion of very LBW babies admitted influenced the outcome since it was strongly linked to the average duration of study, the number of beds and nurses, and also the outcome. In the analysis, these proportions did not correlate with the CFR. This could be because only a small proportion of the admitted babies weighed less than 1,500 g (8.5%). It was also observed that the outcome of babies weighing 1,500-2,499 g improved significantly but that for the very LBW babies the results were not very encouraging.

### Proportion of outborn babies

The CFR increased with the increased proportion of the outborn babies. Almost 45% of the variation in the CFR could be explained by the proportion of the outborn cases availing of SCNU services.

## DISCUSSION

The results of the assessment of the eight units suggest that quality level II newborn care can be provided at the district level within the public-health system. According to the estimates, about 10-15% of all newborns have a complication requiring level II care. In the present study, the proportion was highly skewed. While in some units it was 4%, it touched 40% in others. It was less in units where strict admission criteria were in place or where people preferred to vist private doctors. On the other hand, in the scenario where the surveyed unit was the only facility available for special care, most admissions took place in that hospital.

The number of beds was less than what was required across all the units. Results of many studies were published on the provision of beds in neonatal units from developed countries (9,10). While the number of deliveries taking place in the hospital where the unit is located forms the basis, a number of other parameters, such as average duration of stay, proportion of babies requiring special care, proportion of outborn babies, and proportion of LBW infants form the determining factors. The rule of thumb method based on the guidelines of the National Neonatology Forum considers only the proportion of babies requiring special care and the average duration of stay.

The mean proportion of babies admitted to the SCNUs compared to the number of livebirths was 24.7% (range 14-47%) in Thames in 1975 (11). Not all babies who were admitted needed intensive care. Over one-third of the workload of the typical unit was generated by infants of normal or near normal birthweight who were admitted for a short stay and received no special medical treatment. This is similar to our observations from many surveyed units that, in many instances, the babies were admitted for observation. This increased the workload and the bed occupancy rate, and the quality of care suffered. Experiences from many countries indicate that care gets compromised as a result of admission overload (12).

The admission policy of a unit is also a key indicator that can influence the performance. Except for one unit (Purulia) where it was very stringent, in none of the units these were followed despite having the clear-cut guidelines in place. There was an overdependence on the SCNUs in most places, and in many instances, babies were kept for mere observation due to social pressure. The Purulia SCNU followed very stringent criteria to admit a neonate. If a neonate was admitted to the paediatric ward for over 48 hours, the baby was denied admission despite the availability of beds. A critical observation was that, in none of the SCNUs, except Purulia, neonates were admitted to the paediatric wards. It was, therefore, important to analyze the outcome of admission of neonates in the paediatric wards. But this, unfortunately, was beyond the scope of the study. In Vaishali, on the other hand, many newborns requiring special care ended up in a neighbourhood private hospital as it is the preferred option of the people. Similar observations were noted in Uganda where only infants having complications within 24 hours of birth were admitted to newborn units. Infants who developed complications, such as jaundice, or infection in postnatal wards, or infants discharged from hospital or readmitted to general paediatric wards were not admitted. Babies born at home but who arrived after 24 hours of age and those discharged from the neonatal unit but who later developed complications were not admitted (12). Also, currently, the units had largely inborn admissions, thus considerably leaving a large proportion of sick newborns delivered outside the facilities. If one estimates the numbers of newborns in the district who would require level II care, the numbers of special care beds required would be much larger, indicating a huge unmet needs for special newborn care.

The CFR among the admitted neonates in our study varied from 7% to 18%. The national neonatal perinatal database of 2002-2003 shows that 11.4% of admitted neonates (extramural) expired while it was 0.9% for intramural cases. The database also indicates that the primary causes of deaths were perinatal asphyxia and extreme prematurity in both the groups. Sepsis contributed to 25.2% of mortality among extramural cases while it was 4.1% among inborn admissions (13). These are quite close to the figures obtained in our study. The variations observed across the units in our study are largely due to lack of uniform case definitions being followed in the units.

Most units had shown a decline in the mortality rate within one year of their functioning. Some units, such as Lalitpur and Mayurbhanj, had shown an increase, which could probably be due to lack of adherence to aseptic measures and a huge admission overload in Mayurbhanj. The rise in the mortality rate in Lalitpur could probably be explained by the number of nurses that was halved abruptly. An increase in the CFR indicated that the gains that had been achieved might not be sustainable for various reasons. Factors influencing their performance must be looked into and regularly monitored.

The admission overload was a concern in most units. It must be considered here that the number of beds is a crucial parameter because human resources, equipment, and admission load finally depend on the number of beds. Given the fact that the number of deliveries had increased in most units and the bed occupancy rate exceeded 100%, the number of beds for each unit needs to be revaluated. An increased admission overload also gives rise to sharing of beds often by 2-3 babies which poses a risk. This was a common observation in the districts of Mayurbhanj and Guna. Chances of acquiring infection increases manifold with sharing of beds.

The maintenance of equipment was a major challenge in most districts. The SCNUs were mostly sited in remote, difficult-to-reach locations. The equipment-providing companies had their offices at the state level or sometimes these were not there in every state. Service engineers at the state level generally prepared their roster for their round to attend the complaints but they preferred to plan their route map in a way that the districts falling on a particular route got covered all together, or they had a tendency to wait for the adequate number of complaints from districts on a particular route. This made economic sense to the equipment-providing company but it actually delayed in attending to complaints as by the time the turn of the particular SCNU came in the roster, it had already been quite late. This is a critical issue, and the situation would worsen in near future as the equipment would near their shelf-lives, and the frequency of breakdown would further increase.

Experiences worldwide have shown that level II units can contribute maximally towards bringing down the mortality rate among LBW babies (11,12,14,15). With improved performance in the functioning, the NMR among infants of >1,500 g can match that of a level III unit. In our assessment, this kind of analysis was difficult because of non-availability of sufficient data. Data from Mayurbhanj indicated that the NMR among infants with birthweight of >1,500 g showed a decline while those having very LBW did not benefit much. Similar findings were discussed in India in a level II unit where weights of babies in the range of 1,500-2,499 g had the maximum decline in mortality (14). The impact of level II units on very LBW babies yielded mixed results (12,15).

The SCNU is largely driven by human resources. The optimal number of doctors, nurses, and ancillary staff form the cornerstone to its functioning. In these units, most doctors were transferred from the PHCs. This approach may help address shortages in specific circumstances but are not likely to resolve the problem in the long run. Three of the eight units had a suboptimal bed:nurse ratio which significantly affected the performance of the units. The requirement of the nursing staff in a neonatal nursery depends on the work overload demands (direct and indirect care). Amount of nursing care that an infant needs is somewhat unrelated to how sick that infant is (16). The number of nursing staff is a critical parameter to ensure the quality of care. With the increased beds:nurse ratio, handwashing practices also get compromised to a great extent, directly influencing the quality of care. The Special Care Baby Unit, Kampala in Uganda, lacked nursing staff qualified in neonatal care. Over a period under study (1984-1989), the unit was staffed by 10-12 midwives, and only one midwife caring for 20-30 neonates was a common observation (12). In an investigation carried out in a neonatal special care unit in the USA, the infant:nurse ratio and infant census were the key determinants of nosocomial infections (17). In a neonatal unit in Barbados, the shortage of staff had fostered deterioration in handwashing technique leading to outbreaks of nosocomial infections (18). Maintaining an ideal bed:nurse ratio is a challenge as observed in Uganda, Greece, and Ghana (12,19,20).

To partly overcome the problem of severe shortage of trained nurses for the SCNU in Purulia, the concept of Newborn Aides was developed. Local young women with 10-12 years of school education were given hands-on-training (21). Their performance was evaluated before giving them responsibilities. In the Vaishali SCNU, most nursing work was taken care of by female health workers.

The findings of our study revealed that the CFR strongly correlated with the practices of nurses and doctors in following aseptic precautions. The environmental survey in one of the neonatal units in the UK indicated that transmission of infection was due to inadequate handwashing of nurses and mothers (22). A problem-based and task-oriented education programme has been shown to improve hand-hygiene compliance in Hong Kong. After the intervention, overall hand-hygiene compliance increased from 40% to 53% before patient contact and from 39% to 59% after patient contact. More marked improvement was observed for high-risk procedures. The average number of patient contacts decreased from 2.8 to 18 per patient per hour. The rate of healthcare-associated infection reduced from 11.3 to 6.2 per 1,000 patient-days (23). A statewide hospital-based quality-improvement project targeting hospital staff and community physicians was effective in improving documented newborn preventive services (24). Based on a concept described by the Vermont-Oxford Network, random safety audits were introduced in a level 3 NICU in the UK to improve infection control and routine neonatal care. At the end of six months, compliance with infection-control standards improved from a median of 70% (range 20-100%) to 95% (range 66-100%). This had been recommended strongly to improve clinical practice (25).

The duration of stay in the intensive care units is well-dependent on birthweight. The average duration of stay in an SCNU is usually 5-7 days (4). The average duration of stay for preterm babies or very LBW babies is usually long, and the proportion of LBW babies affects the average duration of stay. It varied between two and 15 days in our assessment. The average stay of patients varied from 26 days at 32-33 weeks to seven days at term, according to a study in New Zealand on level II and III units (26). In California, the average hospital stay for LBW infants ranged from 6.2 days to 68.1 days whereas the average hospital stay for infants who weighed >2,500 g at birth was 2.3 days. Infants who weighed >1,249 g had progressively shorter hospital stay (27).

## Limitations

Although the study was one of the initial ones to give an evidence of feasibility of operating these district-level units, yet it has its own limitations. The information collected was based on secondary data routinely obtained by the respective units triangulated by personal observations. Reporting of morbidities and mortalities is a concern owing to lack of uniform case definitions used in different units. Much of the outcomes could have been related to birthweight and inborn/outborn status but this could not be analyzed because of absence of case-based data. Moreover, data were made available till September 2009. These were compared with data of previous years which may not be very appropriate due to seasonal variations. Although we attempted to find out the possible factors that could be associated with the CFR, it was difficult to come to a meaningful conclusion due to the small sample-size. Despite these limitations, the assessment gives an insight into the potential challenges that might not have been captured during the routine monitoring.

## Conclusions

The SCNUs are a critical investment to curb the neonatal mortality rate in India. Not only these are difficult to establish but it is equally important to maintain their performance. Initial results in the form of reduction in the CFRs are encouraging but there are challenges that need to be looked into before it is scaled up. Having an adequate number of personnel, right policies to facilitate timely repair of equipment, provision of an adequate number of beds, and imparting skills to maintain asepsis are the key recommendations that will circumvent the existing challenges. Replicating such a technically-intensive model, which involves a great deal of coordination and support of various agencies and the acceptability of the implementing authorities and health personnel, is a herculean task. It is pertinent to learn from the experiences, for us to establish the success of this model in diverse settings. It is hoped that lessons learnt from this assessment would assist in scaling up of such units with quality of newborn care facilities in other similar settings.

Different Islamic populations have different alimentary habits, notably during Ramadan. The paper reports the change of diet, lipids, and lipoproteins produced during Ramadan in one Tunisian population. During Ramadan, the study subjects consumed more proteins, cholesterol, vitamin E (p<0.01), and polyunsaturated fatty acids (p<0.05). At the same time, they exhibited an increase in total cholesterol, low-density lipoprotein-cholesterol (p<0.01) and apoprotein B (p<0.05) and a decrease in the ratio of apoprotein AI to apoprotein B (p<0.01). All assayed saturated fatty acids were unaffected by Ramadan fasting while three unsaturated fatty acids (C18:1cis9, C18:2n-6, and C30:4n-6) increased significantly. A return to the habitual diet for a four-week period was not sufficient to restore the pre-fasting patterns. For the study subjects, Ramadan was clearly associated with a change of diet and biochemical profile but its effective impact on atherosclerosis risk was unclear, perhaps, because other non-alimentary changes ought to be considered too. Future studies considering the no-alimentary factors, such as sleep and physical activity, would be useful to clarify the contribution of dietary change in the observed modification of biological profile.

Different Islamic populations have different alimentary habits, notably during Ramadan. The paper reports the change of diet, lipids, and lipoproteins produced during Ramadan in one Tunisian population. During Ramadan, the study subjects consumed more proteins, cholesterol, vitamin E (p<0.01), and polyunsaturated fatty acids (p<0.05). At the same time, they exhibited an increase in total cholesterol, low-density lipoprotein-cholesterol (p<0.01) and apoprotein B (p<0.05) and a decrease in the ratio of apoprotein AI to apoprotein B (p<0.01). All assayed saturated fatty acids were unaffected by Ramadan fasting while three unsaturated fatty acids (C18:1cis9, C18:2n-6, and C30:4n-6) increased significantly. A return to the habitual diet for a four-week period was not sufficient to restore the pre-fasting patterns. For the study subjects, Ramadan was clearly associated with a change of diet and biochemical profile but its effective impact on atherosclerosis risk was unclear, perhaps, because other non-alimentary changes ought to be considered too. Future studies considering the no-alimentary factors, such as sleep and physical activity, would be useful to clarify the contribution of dietary change in the observed modification of biological profile.

## ACKNOWLEDGEMENTS

The work was supported by funding from the UNICEF. The authors acknowledge the contribution of the In-charge of the hospitals and SCNUs in providing data, the district coordinators and project officers of UNICEF who helped retrieve the data from secondary sources. They are also thankful to Ms Alka Chadha, Consultant, PHFI, who helped in data collection, management, and report writing. There is no conflict of interest. PM is employed with the UNICEF. Views presented are of the authors and not of the organizations they represent.
